# Engagement in Prescription Opioid Tapering Research: the EMPOWER Study and a Coproduction Model of Success

**DOI:** 10.1007/s11606-021-07085-w

**Published:** 2021-08-13

**Authors:** Aram Mardian, Luzmercy Perez, Ting Pun, Matthias Cheung, Joel Porter, Korina De Bruyne, Ming-Chih Kao, Pamela Flood, Nathaniel Moore, Luana Colloca, Eric Cramer, Claire E. Ashton-James, Kate Lorig, Sean C. Mackey, Beth D. Darnall

**Affiliations:** 1 Phoenix VA Health Care System, Chronic Pain Wellness Center, Department of Family, Community, and Preventive Medicine, University of Arizona College of Medicine – Phoenix, 650 East Indian School Road, Phoenix, AZ 85012 USA; 2 Department of Anesthesiology, Perioperative and Pain Medicine, Stanford University School of Medicine, 1070 Arastradero Road, Suite 200, MC 5596, Palo Alto, CA 94304 USA; 3 Department of Obstetrics and Gynecology, Queen Mary Hospital, University of Hongkong, Hongkong, China; 4 Thomas J. Long School of Pharmacy, University of the Pacific, Stockton, CA USA; 5 Department of Family Medicine, Intermountain Healthcare, 2075 N University Park BLVD, Layton, UT 84041 USA; 6 Division of Primary, Preventive and Community Care, Stanford School of Medicine, Stanford University School of Medicine, Stanford, CA USA; 7 Department of Anesthesiology, Perioperative and Pain Medicine, Division of Pain Medicine, Stanford University School of Medicine, Stanford, CA USA; 8 MedNOW Clinics, Denver, CO USA; 9 University of Maryland School of Nursing, Baltimore, MD USA; 10 University of Maryland School of Medicine, Baltimore, MD USA; 11 Pain Management Research Institute, The University of Sydney, St Leonards, Australia; 12 Stanford University School of Medicine, Stanford, CA USA

## Abstract

Patients with chronic pain experience stigma within the healthcare system. This stigma is compounded for those taking long-term prescription opioids. Often, public messaging and organizational policies have telegraphed that opioid treatment is a problem to be solved by focusing only on medication reduction efforts. Lack of data has contributed to misperceptions and poor opioid policies. In part, data collection remains poor because patients feel fractured from systems of care and are often not interested in engaging with opioid reduction mandates and research. Similarly, clinicians may fail to engage with opioid stewardship and research due to complexities that exceed their training or capacities. The EMPOWER study applies a *coproduction* model that engages researchers, patients, clinicians, managers, and other health system users. Key stakeholders shaped the design of the study to best ensure acceptability and engagement of the “end users”—patients who enroll in the study *and* the clinicians who implement the opioid tapers. Targeting the needs of any stakeholder group in isolation is suboptimal. Accordingly, we detail the EMPOWER patient-centered opioid tapering clinical research framework and specific strategies to address stakeholder concerns. We also discuss how this framework may be applied to enhance engagement in healthcare research broadly.

**Supplementary Information:**

The online version contains supplementary material available at 10.1007/s11606-021-07085-w.

Patients with chronic pain, particularly those taking long-term opioids, are often marginalized in the healthcare system and in society. Such marginalization can contribute to delayed diagnosis or misdiagnosis, stigma and bias in treatment, barriers to care, and decreased effectiveness of care. Stigma has been amplified by the frequent misperception that continued opioid use indicates an opioid use disorder and by blaming patients with chronic pain for opioid-related harms. A lack of high-quality longitudinal data for opioid use, analgesia effectiveness and functional improvement, opioid misuse, and opioid reduction has perpetuated reductive policies, poor pain care, increased bias and stigma, increased health risks and mortality^1–4^, and fractured patient-clinician relationships and has created barriers to care^5,6^. For instance, some physicians may decline to accept new patients with existing opioid prescriptions. More recently, patients have been further alienated by organizational, state, or federal mandates to taper to pre-defined doses or completely off—often without access to effective therapies to replace the opioids being withdrawn nor support to taper safely. Mandated or administrative opioid taper practices undermine patient agency, and fail to factor patient preferences, medical needs, or response to tapering including worsening functioning. Indeed, the primary focus has been on opioid doses rather than on patient-reported outcomes, further alienating patients who deserve assurances that their safety and pain care are the clinical priorities. Developing evidence-based solutions requires engaging and retaining patients in opioid research, and collecting the real-world data needed to inform better care and policies. However, patient engagement in opioid research remains challenging in this vulnerable and marginalized population.

In clinical research, there is growing interest in engaging researchers, patients, clinicians, managers, and other healthcare system users as coproducers of evidence^7^. This *coproduction* model was developed outside of healthcare^8^ and includes the essential insight that when the providers and consumers of services work collaboratively, they produce greater value^9^. Recently, the coproduction model has been applied to the delivery of healthcare services to improve the quality and costs of healthcare and patient health^10^. As applied to research, the collaborative group process more fully engages research and healthcare teams, and patients. It is associated with a shift from an exclusive notion that research is the sole domain of the academics, to an expanded view that purposefully aligns with the patients’ priorities, goals, and concerns^9^. We apply the coproduction model for research to the EMPOWER study where we focus on both the patient and clinician needs and preferences, and also apply careful attention to the clinician-patient relationship as well.

EMPOWER is a four-state, eleven-clinic pragmatic comparative effectiveness study of two evidence-based behavioral pain treatments applied within the context of a voluntary patient-centered opioid tapering program^11^. The study sites include academic and private pain clinics, primary care clinics, and a Veterans Health Administration site. Opioid reduction can be a fraught topic and patient engagement is predicated on attending to their clinical needs and individual preferences. To ensure patient stakeholder engagement, EMPOWER was conceived in partnership with 250 patients taking prescription opioids who advised us on the study design and our choice of outcome variables. Patients told us they would prefer to be randomized to a behavioral treatment group or not, rather than be randomized to an opioid taper or not (see Table 1). As such, the EMPOWER protocol was designed so that all enrolled patients would engage in a gradual, collaborative opioid taper and patients would be randomized to receive either one of two evidence-based behavioral group treatments (8-session therapist-led cognitive behavioral therapy for chronic pain^12,13^ or 6-session peer-led chronic pain self-management training^14^) or to receive no behavioral treatment (taper only). While the two behavioral treatments have distinctions, they both involve pain education, relaxation training and other pain management skills, and tailoring class content into action plans. Importantly, the process for opioid tapering conforms to the preferences identified by the surveyed patients, and patient choice is supported in the specific mechanics of implementing the collaborative opioid taper. For instance, specifically we found that most patients surveyed said they would be interested in joining an opioid taper study if it was gradual and accounted for their individual needs, would allow them to pause or stop their taper, and would allow for medication increases if needed—all methods we employed in a prior community-based opioid tapering study^15^. Acknowledging that patients’ primary concern about tapering is experiencing increased pain, we integrated methods to minimize likelihood of increased pain (very slow and individualized taper pace, optimizing non-pharmacologic treatments and non-opioid medications, reassurances that opioids may be increased if needed, and communication strategies to minimize nocebo effects). We also selected a co-primary outcome that includes opioid dose *and* pain intensity; pain intensity must not be increased at 12 months to be considered a successful taper. Patient-centered pain care provides flexibility to tailor care to each individual’s unique circumstances. Accordingly, EMPOWER endeavors to help patients achieve their “lowest comfortable dose” at 12 months^16^.

**Table 1:** Patient Inclusion in the Design of the Study

Patient engagement	Goal	Outcome
Internet survey of patients’ tapering perceptions (*N*=250)	Identify patient needs and preferences; tailor methods accordingly	- Participation in tapering is voluntary - Participants randomized to behavioral treatment - Co-primary outcome (pain intensity, opioid dose)
National patient advisory panel (*N*=100)	Ensure patient-facing study materials are acceptable and pleasing to patients	- Study logo selected by patients - EMPOWER acronym created by a patient - Surveys vetted by patients - Website and brochure vetted by patients - Peer-to-peer video vignettes offered on website

Next, we created a national advisory panel comprised of 100 patients with chronic pain taking prescription opioids. Patients were recruited from national chronic pain organizations and community support groups through an electronic mailing list using online advertising and screening surveys, and referrals from pain clinicians in more remote areas (e.g., Alaska). A list of patients interested in chronic pain research opportunities was utilized, as well, to identify potential advisory board members. The panel was selected to ensure representative diversity in race and US geographic region^17^. To enhance patient engagement, the national patient advisory panel advised the research team on public and patient-facing materials to ensure acceptability, utility, and proper messaging. In total, there were seven surveys sent to the panel asking for their feedback on (1) the study’s description; (2) format and language used in the study website which includes patient video vignettes for peer-to-peer communication (see Table 1); (3) branding methods including the title and logo of the study; (4) patient education materials; and (5) the instruments devised to collect data (see Supplemental section for survey samples). To ensure accessibility and a broad outreach across the USA, these seven surveys were electronically distributed and collected through REDCap—the database used to capture their feedback and to process their incentive for their participation. Each member of the patient advisory panel was offered $20 each year for their membership and $10 per survey completed (up to eight surveys per year). In addition to the panel’s early contribution to the study design, the panel will continue to provide advice on study operations throughout the lifecycle of the project and eventually on the public dissemination of study findings.

Steps were taken to address the potential harms associated with opioid tapering and patients’ concerns about being abandoned by medical providers. EMPOWER includes a custom informatics platform and monitoring system (CHOIR^18^) to ensure automated weekly contact with patients and to optimize their safety, sense of security, and connection with their care team throughout the taper. The weekly surveys assess for comfort, pain, opioid withdrawal symptoms, mood, satisfaction with the taper, and other indices of wellbeing, with space allowed for free-text responses. The CHOIR system provides responsive messaging and a virtual safety net. For instance, if pain or depression is increased, a message will acknowledge the symptom or problem, offer compassionate validation, advise on actions they can take, and detail the course of action the clinic will take to address that symptom. In addition to collecting multidimensional patient-reported outcomes, each week patients may provide free-text feedback on their experience. This rich information source enables clinicians to tailor their care plan to patient needs, and provides clinicians with clear data and longitudinal documentation to support their care decisions. Finally, research coordinators provide a point of contact for technical issues with surveys and ensure access to study resources.

Next, methods were developed to address key patient concerns about opioid tapering, including increased pain, no control over the taper, no recourse if pain were to increase during a taper, and being unable to attend multiple behavioral classes if assigned to receive them. While addressing key concerns, EMPOWER methods dually functioned to minimize nocebo and optimize placebo and positive expectations^19^. Table 2 describes common patient concerns/barriers to patient engagement in opioid tapering and the EMPOWER methods used to optimize patient engagement by addressing patient control, choice, and voice.

**Table 2:** Addressing Patient Concerns

Patient concern	EMPOWER study method that addresses the concern
No control over the taper	Patients partner with their clinicians about their taper pace including slowing, pausing or stopping the taper. EMPOWER is designed such that the patient response to their previous dose decrease determines the next step in their taper.
Pain will increase with no relief	- Patients are surveyed weekly so symptoms and discomfort may be addressed promptly. The primary focus on the study is on multidimensional experience/progress vs. opioid doses - EMPOWER clinicians are encouraged to focus on overall patient experience of pain - Communication strategies to reduce nocebo
No ability to reach clinicians	Patient communication is enhanced and automated with CHOIR, immediate responses provide clinical direction.
No voice or recourse	Every survey includes qualitative response fields to capture the patient voice and elements of importance not addressed in our quantitative surveys. Patient satisfaction is directly assessed monthly.
Inability to attend behavioral pain classes to which they are randomized	Behavioral classes may be delivered by telehealth

In order to maximize generalizability and to provide urgently needed guidance to clinicians practicing in real-world situations, EMPOWER was designed as a pragmatic study with minimal exclusion criteria that is embedded into routine clinical care. As such, EMPOWER-trained clinicians implement EMPOWER opioid tapering procedures within in their routine clinical care. For this reason, patient centeredness alone is insufficient for the success of EMPOWER; clinician centeredness is also required. To best engage “end-user” clinicians in the EMPOWER study, our development work identified clinicians’ primary concerns or barriers to engagement (see Table 3). Clinician barriers included lack of training in opioid tapering protocols, concerns communicating with patients about opioid tapering, lack of time and resources to engage in research and provide the required support and follow-up for patients, and how to manage not only patient distress, but their own discomfort during opioid tapering conversations. The EMPOWER study addressed these concerns by offering clinicians additional training, resources, and support including opioid tapering training, manuals, and tools for structuring tapering plans; talking points and language to use with patients introducing the idea of collaborative tapering; automated systems for evaluating and responding to patient-reported outcomes; dedicated study coordinators for each clinical site; and access to professional communication and emotion handling skills webinars and coaching. EMPOWER was designed to attend to not only the needs of patients and clinicians, but also the relationship between the patient and clinician. Indeed, the success of medical care is predicated on listening and providing treatment options for the person with ailments and patient willingness to participate with honesty. Contextual dynamics powerfully steer patient engagement and their health outcomes^20,21^. Stigma and negative experiences have fostered mutual patient-clinician distrust, thus perpetuating the problem. Particularly within a climate of opioid reduction mandates, an authoritative approach to opioid tapering has cultivated patient wariness, priming distrust in the medical system. For clinicians, prior negative or emotionally charged experiences with patients related to opioid prescribing may foster an avoidant approach to patient discussions on this topic. However, clinician avoidance widens the chasm between the clinician and patient, further perpetuating the problem. Similarly, negative messages about opioid use have fostered stigma about the patients who take them.

**Table 3:** Clinician-Centered Strategies That Address Clinician Concerns

Clinician concern	EMPOWER method that addresses the concern
Lack of training around opioid tapering protocols	- Background science and training provided - Core philosophies, principles and supports are standardized - Clinician manual provides a step-by-step guide that is flexible to meet the needs of individual patients - CHOIR Opioid Tapering Tools offers structured tapering plans that may be tailored
Unsure how to approach patients about tapering	- Clinician manual includes talking points and language to engage patients in a conversation about collaborative tapering
Lack of time to engage in opioid tapering research	- Clinician manual includes streamlined screening tools to help clinicians efficiently select the most appropriate treatment pathway, and clinical note templates. - CHOIR system automates data on patient-reported outcomes.
Patient problems during tapering will be overwhelming	- EMPOWER methods are designed to minimize patient discomfort, distress and problems. - Symptoms are assessed weekly by the research team and automated responses help patients know what actions they can take and what actions the clinic will take to address them.
Under-resourced	- Study coordinators manage the research aspects of EMPOWER so clinicians are free to focus on clinical aspects of tapering and pain care.
Ill-equipped to manage patient distress or emotions	- EMPOWER clinicians and their referral sources are offered pre-recorded tapering communication webinars and individualized professional coaching as needed (at no cost to the clinician) to gain essential skills for identifying and responding to patient negative emotions, and managing clinicians’ own discomfort during opioid tapering conversations.

The coproduction model specifically acknowledges that the building and maintenance of the clinician-patient relationship are foundational for success with shared decision-making, patient engagement and receptivity, and patient-centered care^22,23^. Aligning with this model, Table 4 describes EMPOWER strategies designed to enhance the patient-clinician relationship and address the issue of mutual distrust and imbalanced decision-making. The EMPOWER clinician training helps bring balance to the shared decision-making process and foster trust by recognizing and supporting clear boundaries in the context of developing an attitude of partnership with the patient.

**Table 4:** Supporting the Patient-Clinician Relationship

Patient-clinician barrier	EMPOWER method designed to address this barrier
Mutual distrust	- The study materials and program provide a manualized guide, flexibility for individual decision-making, and regular capture of multidimensional data to ensure patients are improving - EMPOWER is comparing two behavioral treatments that emphasize patient agency, autonomy, and symptom self-management (2/3 of enrolled patients will receive either group cognitive behavioral therapy classes or group pain self-management classes). ,^16^]
Imbalanced decision-making	- Shared decision-making regarding the taper and pain care. EMPOWER promotes partnership between patients and clinicians to foster this bond

While the future of healthcare research is leveraging patient centeredness to achieve precision medicine and best patient-centered outcomes, we recognize that this cannot be achieved without addressing the unique community and cultural ecology of each specific research project. The EMPOWER study illustrates how the coproduction model for research engagement can be used to address the challenges of conducting meaningful research to inform complex problems such as chronic pain and long-term opioid therapy. Rather than solely addressing the needs of individual stakeholders such as the patient and the clinician, the coproduction model guided us to also address the patient-clinician relationship. This coproduction model for research engagement can facilitate robust engagement of the research team with the community and all key stakeholders. With a focus on identifying and incorporating the perspective of the consumer and on creating a collaborative relationship between the consumers and producers of research, the coproduction model and the strategies used in the EMPOWER study may provide a useful template to address racial disparities, stigma, and other culturally complex issues in healthcare research.

## Supplementary Information

ESM 1 (PDF 45 kb)

ESM 2 (PDF 44 kb)
