# Supplementary material for: Engagement in Prescription Opioid Tapering Research: the EMPOWER Study and a Coproduction Model of Success
Source: J Gen Intern Med. 2021 Aug 13;37(Suppl 1):113–7. doi: 10.1007/s11606-021-07085-w (PMC8993995; doi:10.1007/s11606-021-07085-w)
Supplement: Supplementary file 1 — (PDF 45 kb) [file 11606_2021_7085_MOESM1_ESM.pdf]

Branding Survey 1

Hello,

Thank you for participating in the National Patient Advisory Panel at the Stanford Division of Pain Medicine! Over the coming months you will be sent a series of surveys that will serve as a sounding board for developing upcoming research materials. You will be compensated (in the form of Amazon gift cards) $10 per survey completed, in addition to $20 per year of participation.

Survey #1: Study Branding
This survey will help our study research team decide on a name for our upcoming study. This name will be used when discussing the study with patients, as well as with general advertising and marketing for the study (logo to be developed as well).

Goals:
• To select a study phrase and/or name that is catchy, positive & short
• To reflect the goals of the study and the participant experience
• To collect unique branding ideas from members of the Advisory Panel

Below is a study overview/refresher overview on what our study is for. After reading the study overview, please give consideration to each of the proposed study names and rank each one by preference (1=Most Preferred, 7=Least Preferred). You will also be given an option to provide comments/suggestions regarding the proposed study names, or to submit a study name of your own, on the next page.

Study Overview:
The clinical trial is for chronic pain patients interested in reducing their opioid medication dosage. We will be comparing the effectiveness of two behavioral treatments for patients while they enter a voluntary patient-centered opioid taper program with their physician:

1. Cognitive Behavioral Therapy for Chronic Pain (8-week class)

2. Chronic Pain Self-Management Program (6-week class)

The two behavioral treatments are expected to reduce the impacts of potential withdrawal symptoms, as well as psychological and social difficulties related to chronic pain during and after the taper program. All participants involved in a patient-centered opioid taper will collaborate with their physician to determine the strategy of their tapering program. The goal of the taper is to achieve the lowest comfortable dose of opioids for each patient, while not increasing the intensity of chronic pain levels.

Please rank your preference of each proposed study name.

		1st CHOICE	2nd CHOICE	3rd CHOICE	4th CHOICE	5th CHOICE	6th CHOICE	7th CHOICE
1)	S.P.O.T.S. (Support During Prescription Opioid Tapering Study)	○	○	○	○	○	○	○
2)	Relief For Life	○	○	○	○	○	○	○
3)	My Relief, My Life	○	○	○	○	○	○	○
4)	Less is More	○	○	○	○	○	○	○
5)	R.O.O.T.S. (Reducing Opioids & Optimizing Therapeutic Success)	○	○	○	○	○	○	○

6)

	O.R.C.A. (Opioid Reduction, Care & Action)	○	○	○	○	○	○	○
7)	Power Up Your Relief	○	○	○	○	○	○	○
