# Supplementary material for: Engagement in Prescription Opioid Tapering Research: the EMPOWER Study and a Coproduction Model of Success
Source: J Gen Intern Med. 2021 Aug 13;37(Suppl 1):113–7. doi: 10.1007/s11606-021-07085-w (PMC8993995; doi:10.1007/s11606-021-07085-w)
Supplement: Supplementary file 2 — (PDF 44 kb) [file 11606_2021_7085_MOESM2_ESM.pdf]

Video Vignettes

Please complete the survey below.

Thank you!

This survey asks for your feedback on video vignettes that will serve as patient education materials for the study.

Please watch the short videos below and answer the related questions.

Video 1

Please answer the following questions related to Video 1 .

		Strongly Disagree	Disagree	Neutral	Agree	Strongly Agree
1)	The video is appropriate for the study	○	○	○	○	○
2)	The video is a good length	○	○	○	○	○
3)	The video adequately addresses questions/concerns patients might have	○	○	○	○	○
4)	The video will be helpful for patients	○	○	○	○	○
5)	The video is engaging	○	○	○	○	○

6) Please provide comments and suggestions for video content & editing, implementation, etc.

____

Video 2

Please answer the following questions related to Video 2 .

		Strongly Disagree	Disagree	Neutral	Agree	Strongly Agree
7)	The video is appropriate for the study	○	○	○	○	○
8)	The video is a good length	○	○	○	○	○
9)	The video adequately addresses questions patients might have	○	○	○	○	○
10)	The video will be helpful for patients	○	○	○	○	○

11) Please provide comments and suggestions for video content & editing, implementation, etc.

____

Video 3

Please answer the following questions related to Video 3 .

		Strongly Disagree	Disagree	Neutral	Agree	Strongly Agree
12)	The video is appropriate for the study	○	○	○	○	○
13)	The video is a good length	○	○	○	○	○
14)	The video adequately addresses questions/concerns patients might have	○	○	○	○	○
15)	The video will be helpful for patients	○	○	○	○	○
16)	The video is engaging	○	○	○	○	○

17) Please provide comments and suggestions for video content & editing, implementation, etc.

____
